# Advancements in Osteosarcoma Therapy: Overcoming Chemotherapy Resistance and Exploring Novel Pharmacological Strategies

**DOI:** 10.3390/ph18040520

**Published:** 2025-04-03

**Authors:** Mahmoud Zhra, Shahid Akhtar Akhund, Khalid S. Mohammad

**Affiliations:** Department of Anatomy, College of Medicine, Alfaisal University, Riyadh 11533, Saudi Arabia; mzahra@alfaisal.edu (M.Z.); sakhund@alfaisal.edu (S.A.A.)

**Keywords:** osteosarcoma, chemotherapy agents, chemotherapy resistance, immunotherapy, novel therapies, targeted therapy

## Abstract

Osteosarcoma is recognized as the most prevalent primary bone malignancy, primarily affecting children and adolescents. It is characterized by its aggressive behavior and high metastatic potential, which often leads to poor patient outcomes. Despite advancements in surgical techniques and chemotherapy regimens, the prognosis for patients with osteosarcoma remains unsatisfactory, with survival rates plateauing over the past few decades. A significant barrier to effective treatment is the development of chemotherapy resistance, which complicates the management of the disease and contributes to high rates of recurrence. This review article aims to provide a comprehensive overview of recent advancements in osteosarcoma therapy, particularly in overcoming chemotherapy resistance. We begin by discussing the current standard treatment modalities, including surgical resection and conventional chemotherapy agents such as methotrexate, doxorubicin, and cisplatin. While these approaches have been foundational in managing osteosarcoma, they are often limited by adverse effects and variability in efficacy among patients. To address these challenges, we explore novel pharmacological strategies that aim to enhance treatment outcomes. This includes targeted therapies focusing on specific molecular alterations in osteosarcoma cells and immunotherapeutic approaches designed to harness the body’s immune system against tumors. Additionally, we review innovative drug delivery systems that aim to improve the bioavailability and efficacy of existing treatments while minimizing toxicity. The review also assesses the mechanisms underlying chemotherapy resistance, such as drug efflux mechanisms, altered metabolism, and enhanced DNA repair pathways. By synthesizing current research findings, we aim to highlight the potential of new therapeutic agents and strategies for overcoming these resistance mechanisms. Ultimately, this article seeks to inform future research directions and clinical practices, underscoring the need for continued innovation in treating osteosarcoma to improve patient outcomes and survival rates.

## 1. Introduction

### 1.1. Overview of Osteosarcoma

Osteosarcoma (OS) is the most common primary bone malignancy, primarily affecting children, adolescents, and young adults [1]. This aggressive tumor typically arises in the metaphysis of long bones, particularly around the knee and shoulder. The incidence of osteosarcoma peaks during periods of rapid skeletal growth, making it particularly relevant in pediatric and adolescent populations [1,2]. Although the exact etiology remains largely unknown, several genetic and environmental factors have been implicated in its development. Notably, hereditary conditions such as Li-Fraumeni syndrome, hereditary retinoblastoma, and Rothmund–Thomson syndrome significantly increase the risk of osteosarcoma [3]. Additionally, environmental factors, including previous radiation exposure and specific chemical exposures, may also contribute to its onset [4].

The clinical presentation of osteosarcoma can vary, but patients often initially present with localized pain and swelling at the tumor site. Several patients are first managed conservatively for suspected injury or trauma with no advancements [5]. As the tumor progresses, systemic symptoms such as fever, weight loss, and fatigue may develop [6]. Radiological imaging techniques, including X-rays, magnetic resonance imaging (MRI), and computed tomography (CT) scans, are essential for diagnosis and staging, assessing tumor size, location, and potential metastasis. Additionally, bone scans can also be employed to detect skeletal involvement outside the primary site [7]. Histopathological examination of biopsy specimens is crucial for confirming the diagnosis, where characteristic patterns of osteoblastic, chondroblastic, or fibroblastic differentiation can be identified [8].

### 1.2. Standard Treatment Modalities

The treatment of osteosarcoma has evolved significantly over the years. The standard approach now involves a combination of surgical resection and chemotherapy, which has been foundational in improving patient outcomes [9]. Surgical intervention typically aims for complete tumor removal, often necessitating limb-salvage procedures or, in some cases, amputation [10]. The introduction of neoadjuvant chemotherapy has transformed the management of osteosarcoma by facilitating tumor shrinkage before surgery, allowing for more conservative surgical approaches and potentially enhancing survival rates [11].

The chemotherapy regimen commonly employed for osteosarcoma includes agents such as methotrexate, doxorubicin, and cisplatin [12]. These drugs work by targeting rapidly dividing cancer cells, but they are not without significant side effects. Adverse effects such as myelosuppression, gastrointestinal disturbances, and cardiotoxicity can severely impact a patient’s quality of life and complicate treatment adherence [13]. Furthermore, the efficacy of these agents varies among patients, with some experiencing robust responses while others exhibit minimal benefit. This variability is a significant barrier to achieving consistent treatment outcomes [14].

### 1.3. The Need for New Treatment Approaches

Despite advancements in surgical techniques and chemotherapy regimens, the prognosis for patients with osteosarcoma remains unsatisfactory, particularly in cases with metastatic disease. The five-year survival rate for localized osteosarcoma is approximately 60–70%, but this figure drops significantly to about 20–30% for patients with metastasis at the time of diagnosis [15,16,17]. A substantial barrier to effective treatment is developing chemotherapy resistance, which complicates the management of the disease and contributes to high rates of recurrence [18]. The mechanisms underlying this resistance are multifaceted and can include drug efflux, enhanced DNA repair mechanisms, and alterations in cellular metabolism [19].

Research has identified several key factors within the tumor microenvironment (TME) that contribute to developing chemotherapy resistance. The TME is a complex ecosystem composed of various cell types, including immune cells, fibroblasts, and endothelial cells, along with extracellular matrix components [20]. Interactions between cancer cells and their microenvironment can influence tumor growth, metastasis, and response to therapy [21]. For instance, cancer-associated fibroblasts (CAFs) can secrete growth factors and extracellular matrix proteins that promote tumor survival and limit the effectiveness of chemotherapy [22]. Additionally, immune cells in the TME can adopt pro-tumorigenic roles, further complicating treatment outcomes [23]. Moreover, adaptive mechanisms of the TME, such as extracellular matrix remodeling and hypoxia adaptation, create additional barriers to effective therapy [24].

To address the challenges posed by chemotherapy resistance, there is a growing interest in novel pharmacological strategies to enhance treatment outcomes for osteosarcoma patients. Targeted therapies focusing on specific molecular alterations present in osteosarcoma cells have emerged as promising alternatives to conventional chemotherapy [25,26]. These therapies exploit the unique vulnerabilities of osteosarcoma cells by targeting the signaling pathways and genetic mutations driving tumor growth [26]. For instance, inhibitors of the mammalian target of rapamycin (mTOR) pathway and receptor tyrosine kinases (RTKs) have shown potential in preclinical and early clinical studies [27,28].

Immunotherapeutic approaches are gaining traction in the treatment of osteosarcoma. By harnessing the body’s immune system, these therapies aim to enhance the antitumor response and overcome the immunosuppressive effects of the tumor microenvironment [29]. Chimeric antigen receptor (CAR) T-cell therapy and immune checkpoint inhibitors have demonstrated promise in other malignancies, and ongoing research is investigating their potential applications in osteosarcoma [30,31,32]. Furthermore, the development of personalized immunotherapies targeting specific tumor antigens represents a rapidly evolving area of research with significant implications for improving patient outcomes [33].

In addition to targeted therapies and immunotherapy, innovative drug delivery systems are being explored to enhance treatment efficacy and minimize toxicity [34]. In particular, nanoparticle-based delivery systems can improve the targeted delivery of chemotherapy drugs to tumor sites, thereby reducing systemic exposure and associated side effects [35]. Various nanocarriers, including liposomes, polymeric nanoparticles, dendrimers, and micelles, have been developed to enhance drug delivery by leveraging the enhanced permeability and retention (EPR) effect for tumor targeting [36,37,38,39]. Furthermore, active targeting schemes, including ligand modifications, enhance the specificity of these nanocarriers [40]. Clinical trials have shown promising results, leading to approvals for formulations like Mepact^®^ [41]. While these advancements significantly improve drug bioavailability and reduce systemic side effects and drug resistance [42], challenges remain in translating these technologies into clinical practice, including issues of nanoparticle stability, scalability, and regulatory hurdles [43,44].

The mechanisms underlying chemotherapy resistance in osteosarcoma are complex and multifactorial. Understanding these mechanisms is critical for the development of effective strategies to overcome resistance [45]. Drug efflux mechanisms, where cancer cells utilize transport proteins to pump out chemotherapeutic agents, can significantly diminish drug efficacy [46,47]. Additionally, alterations in drug metabolism, including increased expression of detoxifying enzymes, can further contribute to resistance [18,48]. Enhanced DNA repair pathways, which allow cancer cells to repair the damage caused by chemotherapy, also play a pivotal role in resistance development [49,50].

We aim to highlight new therapeutic agents’ potential and strategies for overcoming resistance mechanisms by integrating current research findings. Our review provides a comprehensive overview of recent advancements in osteosarcoma therapy, emphasizing the importance of addressing chemotherapy resistance to improve patient outcomes. We will explore the interplay between the tumor microenvironment and treatment efficacy and the role of novel pharmacological approaches in enhancing therapeutic responses.

This article aspires to inform future research directions and clinical practices, underscoring the need for continued innovation in the treatment of osteosarcoma. As we strive to improve patient outcomes and survival rates, a deeper understanding of the molecular underpinnings of osteosarcoma and its microenvironment will be essential in guiding the development of more effective therapies.

## 2. Chemotherapy Resistance in Osteosarcoma: Therapeutic Implications

Osteosarcoma (OS) is the most common primary malignant bone tumor in children and young adults, characterized by high metastatic potential and poor prognosis for metastatic cases [16,51]. Standard treatment for osteosarcoma typically includes a combination of surgical intervention and multi-agent chemotherapy. The surgical approach focuses on removing the tumor with adequate margins while maintaining the functionality of the affected limb [12,52]. Pre-operative and post-operative chemotherapy regimens, which include methotrexate, doxorubicin, and cisplatin (MAP), are designed to reduce tumor size before surgery and to eliminate any residual tumor cells afterward [53].

However, systemic chemotherapy for OS faces significant challenges due to its invasive nature and the pain it causes, which can significantly impact the quality of life of patients [54]. One major issue is that systemic drugs often struggle to reach cancer cells located far from blood vessels, resulting in low drug concentrations in the affected bone. This problem is exacerbated by factors such as drug instability in the bloodstream, protein binding, and clearance by liver cells, which all contribute to toxicity related to the doses given [55]. As a result, high systemic doses are frequently required, which can lead to severe adverse effects, including myelosuppression, hepatotoxicity, cardiotoxicity, and potentially fatal central nervous system complications. Chemotherapy resistance in OS remains a significant obstacle, with over 30% of patients exhibiting resistance to current treatments or experiencing severe side effects, ultimately leading to disease progression and increased mortality [56]. To address these challenges, recent studies have concentrated on developing advanced drug delivery systems aimed at enhancing the effectiveness of chemotherapy while minimizing side effects [57]. Additionally, understanding the mechanisms of chemotherapy resistance is essential for creating new therapeutic strategies to improve patient outcomes.

### 2.1. Mechanisms of Chemotherapy Resistance

**Figure 1:**
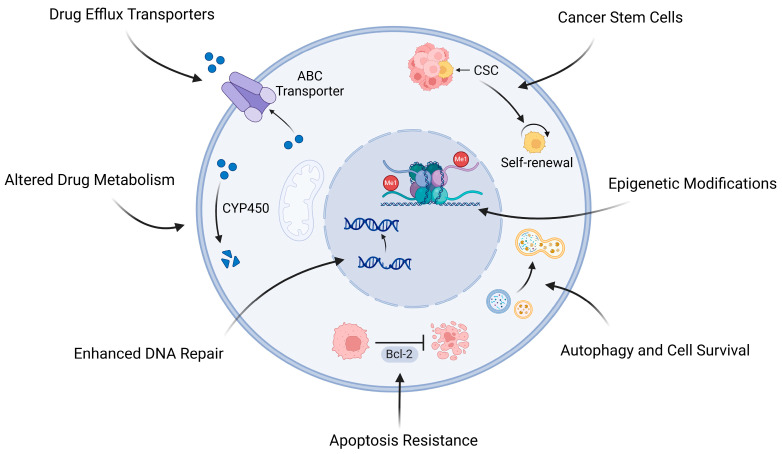
This figure illustrates the key mechanisms contributing to drug resistance in cancer cells. Drug efflux transporters, such as ATP-binding cassette (ABC) transporters, actively expel chemotherapeutic agents, reducing intracellular drug accumulation. Altered drug metabolism, mediated by enzymes like cytochrome P450 (CYP450), modifies and deactivates drugs, decreasing their effectiveness. Cancer cells also exhibit enhanced DNA repair mechanisms, allowing them to fix chemotherapy-induced damage and sustain proliferation. Additionally, apoptosis resistance, facilitated by anti-apoptotic proteins like Bcl-2, enables cancer cells to evade programmed cell death. Autophagy and cell survival pathways further promote resistance by helping cells endure stress conditions, including exposure to chemotherapy. Epigenetic modifications, such as DNA methylation and histone alterations, regulate gene expression in ways that enhance drug resistance. Moreover, cancer stem cells (CSCs), a subpopulation with self-renewal capacity, contribute to tumor recurrence and therapy failure. These interconnected processes collectively enable cancer cells to adapt and survive under therapeutic pressure, posing significant challenges to effective treatment.

Chemotherapeutic resistance is an ongoing problem in osteosarcoma therapy driven by multidimensional biological pathways facilitating survival and adaptation to chemostress in tumor cells. Among these essential pathways is overexpression of ATP-binding cassette (ABC) transporters to actively remove chemotherapeutic drugs from cancer cells; changes in metabolism through enzymes such as cytochrome P450 (CYP) and glutathione S-transferases (GSTs) to modulate inactivation and clearance of drugs; augmented DNA repair pathways, including base excision repair (BER) and nucleotide excision repair (NER) pathways, to reverse therapy-induced DNA damage in tumor cells. Furthermore, avoidance of apoptosis—often by TP53 mutation or overexpression of anti-apoptotic factors—allows malignant cells to sidestep cell death checkpoint impediments. Autophagy is another survival pathway often hijacked by osteosarcoma cells to withstand chemotherapeutic insult. DNA methylation and epigenetic modifications can modulate gene expression to support chemoresistant phenotypes further, while cancer stem cells (CSCs) with high self-renewal capacity and extensive survival pathways add an additional layer to this complexity. Collectively, these interwound pathways reinforce the value of combining therapies to target multiple pathways to obtain superior efficacy and better outcomes in osteosarcoma (Figure 1).

#### 2.1.1. Drug Efflux Transporters

Drug efflux transporters, particularly ATP-binding cassette (ABC) transporters, indeed play a critical role in multidrug resistance (MDR) in cancer, including osteosarcoma (OS), by actively effluxing chemotherapeutic agents from cells [58]. The overexpression of the membrane drug transporter ATP-binding cassette subfamily B member 1 (ABCB1), also known as P-glycoprotein (P-gp) or multidrug resistance protein 1 (MDR1), has been extensively investigated in relation to chemotherapy resistance in osteosarcoma, as reported in various studies [59,60,61]. ABCB1 plays a crucial role in OS chemoresistance to doxorubicin by actively transporting the drug out of osteosarcoma cells, thus reducing its cytotoxic effects. High ABCB1 expression combined with low ABCA1 levels is indicative of resistance to chemotherapy in OS [62].

ABCB1 or P-gp expression is regulated by various pathways, including PI3K/Akt [63,64], PTN/β-catenin [65], and ERRα [66]. These pathways influence P-gp expression through transcriptional and post-transcriptional mechanisms, such as miRNA regulation [67]. For instance, the miR-198/ABCB1 axis is implicated in doxorubicin resistance, where the circular RNA circ_0002060 enhances resistance by sponging miR-198, leading to increased ABCB1 expression [68]. Furthermore, Pleiotrophin (PTN) enhances the expression of ABCB1 by activating the ALK/GSK3β/β-catenin signaling pathway, which contributes to doxorubicin resistance in OS cells [69].

Inhibition of P-gp or its regulatory pathways can sensitize cancer cells to chemotherapy [58]. For instance, luteolin enhances chemosensitivity in osteosarcoma by targeting the PTN/β-catenin/MDR1 axis via miR-384 upregulation [65]. Furthermore, Inhibition of Akt, along with GRP78 suppression, has been demonstrated to decrease P-gp levels and mitigate chemoresistance in OS cells [70]. Additionally, the tumor suppressor PTEN negatively regulates PI3K/Akt signaling, and its loss contributes to chemoresistance [71]. Understanding these regulatory mechanisms is crucial for developing strategies to overcome drug resistance in cancer treatment.

#### 2.1.2. Altered Drug Metabolism

One significant mechanism contributing to the chemoresistance of osteosarcoma (OS) is altered drug metabolism, which involves various metabolic pathways and cellular processes. Cytochrome P450 (CYP) enzymes and glutathione S-transferases (GSTs) play crucial roles in the development of chemoresistance in OS through altered drug metabolism [72,73]. CYP, particularly CYP1A2, CYP3A4, and CYP3A5, influence the pharmacokinetics of OS chemotherapeutics, with doxorubicin upregulating CYP1A2 and CYP3A4, while cisplatin and methotrexate enhance CYP3A4 [72]. Higher expression of CYP1A2 correlates with better event-free survival, while elevated CYP3A4/5 is linked to distant metastasis and poor prognosis, indicating their potential as biomarkers [74]. GTPs, particularly glutathione S-transferase P1 (GSTP1), expression increases in response to doxorubicin and cisplatin treatment of OS [73]. Furthermore, GSTP1 contributes to doxorubicin and cisplatin resistance in OS, potentially through the activation of extracellular signal-regulated kinase (ERK)1/2 [73]. Genetic variations in GSTP1 affect treatment outcomes, with the G/G genotype associated with shorter survival and poorer responses, though links with GSTM1 and GSTT1 polymorphisms are inconsistent [75].

The potential of CYP and GST enzyme inhibitors to enhance chemotherapy efficacy in osteosarcoma is promising due to their roles in drug metabolism and chemoresistance. Researchers are investigating multifunctional drug complexes that integrate chemotherapeutic agents with GST inhibitors. For example, platinum (IV) complexes containing GST inhibitors have been developed to address cisplatin resistance in osteosarcoma treatment [76,77]. Furthermore, NBDHEX (6-(7-nitro-2,1,3-benzoxadiazol-4-ylthio)hexanol) and its analogs are promising GST inhibitors, mainly targeting GSTP1. They activate the JNK/c-Jun signaling pathway, dissociating the TRAF2-GSTP1 complex. It has been proposed as a potential treatment for cisplatin-resistant human osteosarcoma [78].

Additionally, CYP3A4 inhibitors such as ketoconazole may help overcome chemoresistance in OS by enhancing the efficacy of chemotherapy. Although direct studies in OS are limited, findings from other cancers suggest potential benefits [79,80,81]. For instance, the pregnane X receptor (PXR) is involved in drug metabolism and resistance in OS [82]. Ketoconazole disrupts the interaction between PXR and HNF4α, which is essential for activating the CYP3A4 promoter. This disruption reduces CYP3A4 expression and the metabolism of chemotherapeutic agents, leading to increased intracellular concentrations and enhanced cytotoxic effects [83,84].

#### 2.1.3. Enhanced DNA Repair Mechanisms

Chemotherapeutic drugs induce DNA damage to kill cancer cells, but tumor cells can enhance DNA repair pathways to resist treatment [85]. The DNA damage response (DDR) plays a crucial role in maintaining genome stability and chemoresistance [86]. Base excision repair (BER) is a crucial DNA repair mechanism that maintains genome integrity by repairing thousands of DNA lesions caused by endogenous and exogenous mutagens [87]. In osteosarcoma (OS), BER plays a significant role in chemotherapy resistance and cancer progression [86]. The BER pathway involves multiple steps, including base excision, strand incision, end processing, gap filling, and nick sealing, carried out by various enzymes such as DNA glycosylases, AP endonucleases, polymerases, and ligases [88,89]. PARP1 (Poly (ADP-ribose) polymerase 1) plays a crucial role in the BER pathway, where it detects single-strand breaks (SSBs) in DNA and recruits repair proteins to facilitate damage repair [90]. Knockdown of PARP1 has been found to inhibit proliferation and increase chemotherapy sensitivity to doxorubicin in osteosarcoma cells [91].

Several studies have identified genetic variations in the DNA repair gene, excision repair cross-complementation (ERCC), part of the nucleotide excision repair (NER) pathway, that influence the chemotherapy resistance and survival rates in osteosarcoma patients [92,93,94,95,96]. Elevated levels of ERCC proteins are associated with resistance to cisplatin. Igarashi et al. reported that the overexpression of ERCC1 correlates with resistance to cisplatin-based chemotherapy in osteosarcoma [97].

The application of DNA repair inhibitors has emerged as a promising strategy to combat chemotherapy resistance, as inhibiting specific DNA repair pathways may increase chemotherapy effectiveness [98]. For instance, Inhibitors targeting nucleotide excision repair (NER) and base excision repair (BER) pathways and correlate with ERCC, such as NSC130813 and triptolide, have shown promise in overcoming cisplatin resistance in osteosarcoma cells [99]. Recent studies have identified potential targets to overcome chemoresistance in OS, including PAXX, a factor in non-homologous end joining (NHEJ), a key DNA repair pathway, by using a molecule called M11 that can disrupt the PAXX-Ku70 interaction and re-sensitize the chemoresistant osteosarcoma cells to doxorubicin and cisplatin [100].

PARP1 is considered a potential target for overcoming chemotherapy resistance, as it enhances the repair of DNA-damaged cells by recruiting DNA damage response proteins, such as γH2AX and BRCA1/2, which are linked to reduced survival rates in osteosarcoma patients [101]. PARP inhibitors, such as olaparib and talazoparib, have shown potential in enhancing the efficacy of chemotherapeutic agents like doxorubicin and temozolomide in OS cells, offering a potential strategy to mitigate resistance [101,102]. Overall, modulating DNA damage response pathways may provide promising approaches to overcome chemoresistance in OS [103].

#### 2.1.4. Apoptosis Resistance

Apoptosis resistance in osteosarcoma (OS) is one of the main obstacles in osteosarcoma treatment, mainly because of the aberrant regulation of apoptosis pathways. One of the main mechanisms of this resistance is mutations in the TP53 gene and over-expression of anti-apoptotic protein Bcl-2 [104]. TP53 mutations are common in many cancers, including osteosarcoma, causing apoptosis inhibition and malignant cell survival [105]. In OS, the p53 R273H mutant of TP53 is correlated with reduced procaspase-3 (PC-3) expression and ineffectiveness of methotrexate and doxorubicin to induce apoptosis [106]. The Bcl-2 family of proteins is involved in apoptosis regulation, and overexpression of anti-apoptotic proteins, such as Bcl-2 and Bcl-xL, has been associated with chemoresistance [107,108]. For example, circular RNA UBAP2 (circUBAP2) was found to increase the expression of the anti-apoptotic protein Bcl-2. This suggests that circUBAP2 promotes cell survival by enhancing Bcl-2 levels, thereby preventing apoptosis in osteosarcoma cells. Furthermore, the knockdown of circUBAP2 expression inhibited the expression of Bcl-2 and significantly promotes apoptosis in osteosarcoma cells (MG63 and U2OS) [108].

Besides genetic mutations, osteosarcoma’s intrinsic apoptotic pathway is frequently affected. The balance between pro-apoptotic proteins, such as Bax, and anti-apoptotic proteins, such as Bcl-2, primarily regulates this pathway. Overexpression of Bcl-2 has an inhibitory effect on the activation of caspases, which are necessary for the carrying out of the apoptotic program [109,110]. For instance, the application of the compounds diosmetin and 6-gingerol to osteosarcoma cells has been reported to suppress Bcl-2 and augment Bax expression and, therefore, induce apoptosis [109,111]. In addition, PI3K/Akt signaling pathway is frequently involved in the survival of OS cells, in which its activation can result in up-regulation of Bcl-2 and down-regulation of apoptosis [110,112].

To overcome apoptosis resistance, pro-apoptotic agents and BH3 mimetics are attracting interest. These agents are designed to recover their apoptotic signaling cascades, which are frequently impaired in cancer cells. For instance, BH3 mimetics are designed to inhibit the function of anti-apoptotic Bcl-2 proteins, thereby promoting the activation of pro-apoptotic factors and facilitating cell death [113]. Research has demonstrated that compounds like plumbagin and honokiol can induce apoptosis in OS cells through various mechanisms, including the activation of mitochondrial pathways and the generation of reactive oxygen species (ROS) [114,115]. In addition, inhibition of the PI3K/Akt pathway by agents such as apatinib can increase the sensitivity of OS cells to chemotherapy by inducing apoptosis [112,116]. Targeting apoptosis resistance mechanisms in osteosarcoma, such as using BH3 mimetics or disrupting mitochondrial function, may improve treatment efficacy and patient outcomes.

#### 2.1.5. Autophagy and Cell Survival

Autophagy is an essential cellular mechanism that can degrade and recycle various cellular components and has multiple roles in cancer biology. Under the condition of chemoresistance, autophagy is known as a significant pathway through which osteosarcoma (OS) cells escape the inhibitory effects of cytotoxic drugs [117]. Studies have shown that autophagy is upregulated in osteosarcoma cells exposed to chemotherapeutic drugs, such as doxorubicin and cisplatin, allowing these cells to survive under stress conditions [118,119]. For example, autophagy induction via the Beclin-1 pathway has been associated with enhanced doxorubicin resistance, suggesting autophagy functions as a drug-resistance mechanism that prevents death by apoptosis [120].

In addition, microRNAs (miRNAs) have been reported to regulate autophagy and chemoresistance in osteosarcoma. For instance, miR-30a downregulation has been reported to promote autophagy and thereby mediate chemoresistance [120]. On the contrary, miR-410 has been shown to downregulate autophagy, making expression regulation of miRNA a potential sensitizing strategy for osteosarcoma cells to chemotherapy [121]. This crosstalk between autophagy and miRNA regulation explains how autophagy contributes to cancer cell survival and drug resistance complexity.

Therapeutic implications of autophagy inhibitors, including chloroquine and related compounds, are of interest as an approach to overcoming chemoresistance in osteosarcoma. Lysosomal function inhibition by chloroquine reduces autophagic degradation and apoptosis of cancer cells [122]. Moreover, chloroquine has been reported to block the autophagic process in cisplatin-resistant OS cells, thereby enhancing the cytotoxic effects of cisplatin. Evidence for using autophagy inhibitors in combination with existing chemotherapeutic agents has successfully improved global therapeutic efficiency. For example, inhibition of autophagy using 3-methyladenine (3-MA), an autophagy inhibitor, together with mTOR inhibitors like rapamycin has been demonstrated to promote apoptosis in OS cells, which indicates that targeting autophagy may enhance response to therapy [123,124]. In contrast, 3-MA can sensitize OS cells to the mTOR inhibitor, and accordingly, autophagy has been proposed to be a master endpoint for chemoresistance [116,124]. Autophagy is crucial in osteosarcoma chemoresistance, promoting cell survival during treatment. Modulating autophagy and employing inhibitors may enhance chemotherapy sensitivity, improving patient outcomes.

#### 2.1.6. Epigenetic Modifications

Epigenetic modifications play a crucial role in developing chemotherapy resistance in osteosarcoma (OS), primarily through mechanisms involving DNA methylation and histone modifications. Aberrant DNA methylation patterns have been implicated in OS pathogenesis, particularly hypermethylation of tumor suppressor genes (TSG) and hypomethylation of oncogenes [125,126]. For instance, the overexpression of DNMT1 (DNA methyltransferase 1) has been shown to correlate with increased resistance to apoptosis in OS cells, indicating that aberrant DNA methylation patterns are a significant factor in chemoresistance [127]. Furthermore, histone modifications, such as acetylation and methylation, are critical in regulating gene expression related to drug resistance. Histone deacetylases (HDACs) have been implicated in maintaining cancer stem cell properties and promoting resistance to chemotherapy [128,129]. Specifically, the depletion of HDAC2 has been associated with enhanced stemness in OS, suggesting that histone modifications can influence the tumor’s response to chemotherapy [129]. Moreover, in OS, histone H3 lysine 27 trimethylation (H3K27me3) levels are associated with cisplatin sensitivity, with higher levels increasing chemosensitivity [130].

The application of epigenetic drugs, such as DNA methyltransferase and HDAC inhibitors, has emerged as a promising strategy to overcome chemotherapy resistance in OS. For example, the use of HDAC inhibitors like panobinostat has been shown to induce apoptosis and alter the expression of genes involved in drug resistance pathways, thereby enhancing the sensitivity of OS cells to chemotherapeutic agents [131]. Additionally, combining epigenetic drugs with traditional chemotherapy has been suggested to improve treatment outcomes. Studies have indicated that inhibiting both DNA methylation and histone deacetylation can synergistically enhance the efficacy of standard chemotherapeutics like doxorubicin and cisplatin [132,133]. This approach aims to reverse the epigenetic alterations contributing to drug resistance, thereby restoring osteosarcoma cells’ sensitivity to chemotherapy.

Moreover, the interplay between various epigenetic modifications and non-coding RNAs, such as long non-coding RNAs (lncRNAs), further complicates the landscape of chemotherapy resistance in cancer [134]. For instance, lncRNAs have been shown to interact with epigenetic regulators, influencing the expression of genes associated with drug resistance [135]. The lncRNA LINC00161 has been implicated in cisplatin-induced apoptosis, where it attenuates osteosarcoma chemoresistance by targeting the miR-645-IFIT2 signaling axis [136]. Overall, the identification of specific lncRNA signatures that correlate with treatment response could provide valuable biomarkers for predicting chemotherapy outcomes in OS patients [135,137].

#### 2.1.7. Cancer Stem Cells

Cancer stem cells (CSCs) are essential in initiating and progressing osteosarcoma (OS) and significantly contribute to chemotherapy resistance. These cells are characterized by the overexpression of specific markers, including OCT4, SOX2, and NANOG, which are associated with stemness and drug resistance mechanism [138]. For instance, Cavalcanti et al. highlight that patient-derived OS cells exhibit resistance to methotrexate due to the presence of CSCs that express drug transporters like ABCG2 [139]. Similarly, Liu et al. state that the intrinsic properties of CSCs are implicated in the treatment failure of OS, underscoring their role in limiting the effectiveness of conventional chemotherapy [140]. The mechanisms of CSC-mediated drug resistance in osteosarcomas are complex and involve a wide variety of different mechanisms. Wang et al. demonstrated that the EID3 protein enhances the stem-like characteristics of osteosarcoma cells through the activation of the PI3K-AKT signaling pathway, which is known to promote cell survival and chemoresistance [141]. Furthermore, the interplay between various signaling pathways such as Notch, Wnt/β-Catenin, and Hedgehog has been implicated in regulating the self-renewal and survival of CSCs in OS [138].

Targeting CSCs presents a promising non-invasive therapeutic approach to improve chemotherapy resistance in osteosarcoma. Numerous studies have reported that targeting the Hedgehog signaling pathway can lead to reduced tumor growth and increased sensitivity to chemotherapeutic agents [142,143,144]. For example, the Hedgehog pathway inhibitor, vismodegib, has been demonstrated to block the signaling that supports the survival and proliferation of stem cells [142]. Moreover, a study showed that the Hedgehog/GLI1 signaling pathway plays a critical role in regulating cisplatin resistance in OS, suggesting that inhibiting this pathway could enhance the efficacy of existing chemotherapeutic regimens [144].

### 2.2. Current Therapeutic Approaches to Overcome Resistance

The management of osteosarcoma (OS) faces significant challenges due to chemotherapy resistance, which dramatically impacts treatment outcomes and patient survival. Current therapeutic strategies to overcome resistance in OS include combination chemotherapy regimens, dose intensification, and novel drug delivery systems. These strategies aim to enhance the efficacy of existing treatments and introduce new modalities to improve patient outcomes. The following section thoroughly explores these strategies, highlighting their potential and limitations.

#### 2.2.1. Combination Chemotherapy Regimens

The current therapeutic landscape for osteosarcoma (OS) predominantly involves combination chemotherapy regimens, which are essential in addressing the challenges posed by drug resistance. The standard treatment protocol typically includes agents such as doxorubicin (adriamycin), methotrexate, and cisplatin, which have been shown to significantly improve survival rates when used in conjunction with surgical interventions [53]. This approach is necessitated by the multifaceted nature of OS, which often exhibits heterogeneity and varying responses to single-agent therapies. By employing multiple agents, clinicians aim to target different pathways involved in tumor growth and survival, thereby enhancing the overall therapeutic effect and minimizing the risk of resistance [140].

Combination chemotherapy regimens have evolved over the years, with the MAP (methotrexate, doxorubicin, and cisplatin) and IAP (ifosfamide, doxorubicin, and cisplatin) regimens being among the most widely adopted [53,145]. These regimens have demonstrated efficacy in both neoadjuvant and adjuvant settings, improving event-free survival rates in patients with localized OS. For instance, studies indicate that the MAP regimen is associated with a 5-year survival rate of approximately 60–70% in patients without metastasis at diagnosis [146,147]. However, the effectiveness of these regimens can be compromised by the development of chemotherapy resistance, which remains a significant hurdle in treatment [148].

Clinical outcomes from various combination strategies have yielded mixed results, with some regimens showing promise in overcoming resistance. A study on OS chemotherapy highlights the success of multidrug regimens. The T12 Protocol, which includes adriamycin, bleomycin, cyclophosphamide, dactinomycin, methotrexate, and cisplatin, demonstrates significant improvements in progression-free survival (PFS) and overall survival, especially when used with ifosfamide or vincristine. The ABCDMP regimen, which includes the same drugs as the T12 Protocol, also shows notable efficacy, particularly when paired with ifosfamide. Other effective combinations include ABCDMPL (adding vincristine) and AP (adriamycin and cisplatin). The study concludes that these multidrug approaches lead to better survival outcomes for OS patients compared to single-agent therapies [149]. At the same time, EURAMOS-1 trial found that adding ifosfamide and etoposide to the MAP regimen for patients with ≥10% viable tumor did not improve event-free survival and increased toxicity, indicating that such additions may not benefit poorly responding OS patients [150].

In chemotherapy-resistant OS models, the combination of doxorubicin and cisplatin, when paired with novel agents such as olaratumab, has proven effective, particularly as it targets specific pathways involved in tumor growth [151]. Furthermore, incorporating agents like bortezomib has been explored, as it sensitizes OS cells to doxorubicin-induced apoptosis by activating specific signaling pathways [152]. Moreover, combining curcumol with cisplatin has demonstrated synergistic effects in preclinical models by inhibiting M2-like polarization of tumor-associated macrophages, which may contribute to chemoresistance [153]. Additionally, the combination of docetaxel and gemcitabine has been evaluated for its efficacy in treating relapsed and refractory OS, showing improved response rates, particularly in older patients who may be more susceptible to chemotherapy-related toxicities [154]. In a patient-derived orthotopic xenograft (PDOX) model, the combination of temozolomide and irinotecan demonstrated significant efficacy in regressing cisplatin-resistant OS, suggesting a potential treatment strategy for patients with relapsed disease [155].

These findings underscore the potential for combination therapies to enhance the efficacy of existing chemotherapeutic agents and improve patient outcomes. They also highlight the need to carefully evaluate treatment strategies to maximize efficacy and minimize adverse effects.

#### 2.2.2. Dose Intensification and Modification

Dose intensification and modification are crucial in managing osteosarcoma (OS), particularly in addressing the significant resistance that OS exhibits to conventional chemotherapy regimens. OS exhibits significant resistance to conventional chemotherapy regimens, necessitating the exploration of high-dose chemotherapy protocols and careful modification of treatment strategies to enhance therapeutic efficacy while managing toxicity. The standard chemotherapy regimen typically includes high-dose methotrexate (HDMTX), doxorubicin, and cisplatin, which have been shown to improve survival rates in patients with OS significantly [156,157]. The EURAMOS-1 protocol highlights the importance of histologic response to neoadjuvant chemotherapy as a survival predictor, making it essential for treatment planning. Research shows that a higher number of high-dose methotrexate (HDMTX) doses is linked to improved histologic response and survival outcomes, highlighting the need for dose intensification to achieve better therapeutic results [158,159].

High-dose chemotherapy protocols, particularly those incorporating HDMTX, are pivotal in addressing the chemoresistance observed in OS. The administration of HDMTX, often in combination with agents like doxorubicin and cisplatin, has been associated with increased tumor necrosis and improved overall survival rates [160]. However, these high-dose regimens are not without risks; they can lead to significant hematological complications such as thrombocytopenia and leukopenia, which complicate treatment and may necessitate dose modifications [161].

The impact of dose intensification is, thus, a double-edged sword; while higher doses can lead to better tumor control, they also increase the risk of adverse effects, potentially resulting in treatment delays or dose reductions. Consequently, balancing therapeutic efficacy with toxicity is a critical consideration in the management of osteosarcoma [161,162,163]. This balance is essential to optimize treatment outcomes while minimizing the risks associated with high-dose chemotherapy protocols.

#### 2.2.3. Novel Drug Delivery Systems

Novel Drug Delivery Systems (NDDS) play a crucial role in overcoming chemotherapy resistance in osteosarcoma (OS). The challenges posed by multidrug resistance (MDR) in OS necessitate innovative strategies to enhance drug efficacy and minimize systemic toxicity. NDDS can bypass drug efflux mechanisms, control drug release, and disturb tumor metabolism to combat MDR [164]. Among these strategies, nanoparticle-based delivery systems and liposomal formulations have emerged as promising approaches, particularly through targeted drug delivery mechanisms.

Nanoparticle-based delivery systems have shown significant potential in addressing the limitations of conventional chemotherapy. These systems can improve drug solubility, enhance drug accumulation at tumor sites, and reduce systemic side effects through the enhanced permeability and retention (EPR) effect [165,166]. For instance, studies have demonstrated that nanoparticles can effectively deliver chemotherapeutic agents like doxorubicin directly to OS cells, thereby increasing the local concentration of the drug while reducing side effects [167]. Various nanocarriers, including polypeptide nanoparticles [168], biomimetic nanoparticles [169], and liposomes [170], have been developed to improve drug delivery. These nanocarriers can protect drugs from rapid clearance, prolong circulation time, and increase drug concentration at tumor sites [171]. Some nanoparticles are designed to respond to specific stimuli, such as pH [168] or reactive oxygen species [172], enabling controlled drug release. Furthermore, the use of hybrid nanoparticles, such as lipid–polymer hybrids, has been reported to synergistically enhance the anticancer effects of drugs like doxorubicin and edelfosine against drug-resistant osteosarcoma [173].

Liposomal formulations represent a significant advancement in the targeted delivery of chemotherapeutics for osteosarcoma treatment. These lipid-based carriers can encapsulate drugs, protecting them from degradation and facilitating controlled release [174]. Several liposomal formulations have been developed and tested in clinical settings for osteosarcoma treatment. For example, Doxil (liposomal doxorubicin) has shown improved efficacy and reduced cardiotoxicity compared to free doxorubicin in various cancers, including osteosarcoma [40]. Liposomes can be engineered to target specific receptors overexpressed in OS cells, such as CD44, which is associated with drug resistance [175,176]. By modifying liposomal formulations to include targeting ligands, researchers have been able to enhance the uptake of chemotherapeutic agents by OS cells, thereby overcoming some of the barriers posed by MDR mechanisms [177,178].

Targeted drug delivery mechanisms are pivotal in enhancing the therapeutic efficacy of NDDS in osteosarcoma treatment. These mechanisms can be classified into passive and active targeting [179]. Passive targeting relies on the enhanced permeability and retention (EPR) effect, where nanoparticles accumulate in tumor tissues due to their leaky vasculature [180]. Active targeting, on the other hand, involves the functionalization of nanoparticles with specific ligands that bind to receptors on the surface of cancer cells, facilitating increased internalization of the drug [40]. For example, the use of folate receptor-targeted nanoparticles has been shown to significantly improve the delivery of doxorubicin to OS cells, leading to enhanced cytotoxicity and reduced drug resistance [173,181]. Furthermore, nanocarriers, such as micelleplexes, have been developed for the active targeting of OS cells to overcome multidrug resistance (MDR) and non-specific toxicity by incorporating targeting ligands that selectively bind to OS cell receptors, thereby enhancing drug delivery efficacy and reducing systemic toxicity [182]. Additionally, targeting the Ras/Akt/mTOR and Ras/ERK1/2/HIF-1α pathways with self-assembling nanoparticles encapsulating zoledronic acid (NZ) can simultaneously upregulate ABCA1 and downregulate ABCB1, thereby restoring drug sensitivity in doxorubicin-resistant osteosarcoma [62].

The integration of novel drug delivery systems, particularly nanoparticle-based and liposomal formulations, into osteosarcoma treatment regimens, holds great promise for overcoming chemotherapy resistance. By leveraging targeted drug delivery mechanisms, these systems can enhance drug accumulation at tumor sites, improve therapeutic outcomes, and potentially reduce the incidence of systemic side effects associated with traditional chemotherapy.

## 3. Targeted Therapies Based on Molecular Abnormalities

### 3.1. Genetic and Molecular Targets in Osteosarcoma

The molecular landscape of osteosarcoma is complex, with receptor tyrosine kinases (RTKs) such as IGF-1R, PDGFR, and HER2 playing pivotal roles in tumor growth and progression. These RTKs are often overexpressed in osteosarcoma, making them critical targets for therapeutic intervention.

#### 3.1.1. Receptor Tyrosine Kinases (RTKs)

The insulin-like growth factor 1 receptor (IGF-1R) is frequently overexpressed in osteosarcoma, contributing to enhanced cell proliferation and survival through the activation of downstream signaling pathways, including the PI3K/Akt and MAPK pathways [183]. This overexpression correlates with poor clinical outcomes, as it promotes tumorigenesis and resistance to conventional therapies [184,185]. Targeting IGF-1R with specific inhibitors has shown promise in preclinical studies, suggesting that it may serve as a viable therapeutic target in osteosarcoma treatment [186]. However, IGF-1R inhibitors have failed in clinical trials due to the complex interactions of IGF-1R with adhesion receptors and the tumor microenvironment, as well as the insufficient understanding of its role in tumor-associated immune and stromal cells [187,188], although some early-phase trials showed evidence of response, particularly in sarcomas [189].

Similarly, the platelet-derived growth factor receptor (PDGFR) is implicated in osteosarcoma pathogenesis. Studies have demonstrated that PDGFRα and PDGFRβ are expressed in a significant percentage of osteosarcoma samples, and their activation is associated with increased tumor growth and metastasis [190]. Inhibition of PDGFR signaling has been explored as a therapeutic strategy, with agents like imatinib mesylate showing anti-proliferative effects on osteosarcoma cells in vitro and in vivo [191]. However, clinical trials have indicated limited efficacy, suggesting that alternative pathways may compensate for PDGFR inhibition [184].

HER2, another RTK, has also been identified as an important player in osteosarcoma. Overexpression of HER2 has been linked to aggressive tumor behavior and poor prognosis [185]. Targeting HER2 with monoclonal antibodies or small molecule inhibitors may provide a therapeutic avenue, particularly for patients with HER2-positive tumors [186]. The integration of HER2-targeted therapies with existing treatment modalities could enhance therapeutic efficacy and improve patient outcomes.

In addition to targeting these RTKs, the development of RTK inhibitors represents a promising strategy in osteosarcoma management. Recent advances in molecular biology have facilitated the identification of novel RTK inhibitors that can selectively target aberrant signaling pathways in osteosarcoma cells [190]. The use of combination therapies that include RTK inhibitors alongside traditional chemotherapy may also enhance treatment responses and reduce the likelihood of resistance [192].

#### 3.1.2. Multi-Kinase Inhibitors (MKIs)

Multi-Kinase Inhibitors (MKIs) are emerging as promising therapeutic agents in the treatment of osteosarcoma (OS) due to their ability to target multiple pathways implicated in tumor growth and resistance mechanisms [193]. OS is characterized by complex genetic alterations and dysregulation of multiple signaling pathways, including PI3K/Akt/mTOR, JAK/STAT, Wnt/β-catenin, Hippo, Notch, PD-1/PD-L1, MAPK, and NF-κB [25,194]. MKIs target multiple kinases involved in tumor growth, metastasis, and drug resistance. MKIs have shown promise in targeting various kinases in osteosarcoma treatment. These include receptor tyrosine kinases like VEGFRs and RET, which are considered key targets [195]. Cell cycle kinases such as CHKs, CDKs, PLKs, and AURKs are also potential therapeutic targets [196]. Specific kinases identified as crucial for osteosarcoma cell survival include Mirk [197] and PLK1 [198].

Several MKIs have demonstrated efficacy in preclinical models and clinical trials [193]. For example, regorafenib, a potent MKI, has shown regression in patient-derived orthotopic xenograft models and outperformed other MKIs while also demonstrating regression in drug-resistant osteosarcoma models [193]. Furthermore, regorafenib, sorafenib, apatinib, and cabozantinib target multiple receptor tyrosine kinases, including VEGFRs and RET, which are considered key targets in osteosarcoma treatment and have shown clinical benefit in phase II trials [199]. Sorafenib inhibited tumor growth, angiogenesis, and metastasis in preclinical models by targeting ERK1/2, MCL-1, and ERM pathways [200]. Combining sorafenib with everolimus enhanced its antitumor activity by completely inhibiting the mTOR pathway [201]. Cabozantinib inhibits OS cell proliferation and migration while also modifying the bone microenvironment [202]. While these MKIs show promise, larger-scale trials are needed to validate their efficacy and identify predictive biomarkers for response [27,203]. Furthermore, MKIs such as anlotinib, apatinib, and sorafenib have improved progression-free survival in advanced osteosarcoma; however, overall survival remains unchanged due to the rapid development of acquired drug resistance in osteosarcoma [204].

#### 3.1.3. Signal Transduction Pathways

The PI3K/Akt/mTOR signaling pathway plays a critical role in the pathogenesis and progression of osteosarcoma. Dysregulation of this pathway is frequently observed in osteosarcoma, leading to enhanced cell proliferation, survival, and metastasis. The activation of PI3K leads to the phosphorylation of Akt, which subsequently activates mTOR, a central regulator of cell growth and metabolism. This cascade of events contributes to the aggressive nature of osteosarcoma by promoting tumor cell proliferation and inhibiting apoptosis [205,206].

Studies have shown that the aberrant activation of the PI3K/Akt/mTOR pathway is a pivotal event in osteosarcoma development. For instance, Jin et al. demonstrated that silencing GPNMB, a glycoprotein associated with tumor progression, suppressed osteosarcoma cell proliferation and metastasis by inhibiting this pathway [205]. Similarly, Zheng et al. reported that AIM2, a DNA-binding protein, could inhibit osteosarcoma cell proliferation and promote apoptosis by inactivating the PI3K/Akt/mTOR signaling pathway [207]. Furthermore, the involvement of this pathway in epithelial–mesenchymal transition (EMT) has been highlighted, indicating its role in enhancing the invasive potential of osteosarcoma cells [208,209].

Therapeutic agents targeting mTOR have emerged as promising strategies for treating osteosarcoma. mTOR inhibitors, such as rapamycin and its analogs (e.g., everolimus), have shown potential in preclinical studies and clinical trials. For instance, Gupte et al. identified dual inhibition of PI3K and mTOR as a conserved therapeutic vulnerability in osteosarcoma, suggesting that targeting this pathway could enhance treatment efficacy [210]. Moreover, the combination of mTOR inhibitors with other agents, such as zoledronic acid, has been shown to overcome resistance to conventional therapies, further supporting the therapeutic potential of mTOR inhibition [211].

In addition to mTOR inhibitors, other therapeutic agents targeting the PI3K/Akt/mTOR pathway have been investigated. For example, the dual PI3K/mTOR inhibitor NVP-BEZ235 was found to inhibit osteosarcoma cell proliferation and improve survival rates in vivo [191]. Other studies have explored the effects of natural compounds, such as honokiol and alantolactone, which induce apoptosis and autophagy in osteosarcoma cells through the inhibition of the PI3K/Akt/mTOR pathway [110,115]. These findings underscore the importance of targeting the PI3K/Akt/mTOR signaling pathway as a viable therapeutic strategy in osteosarcoma management.

#### 3.1.4. MicroRNA-Based Therapies

MicroRNA (miRNA)-based therapies have emerged as a promising avenue for the modulation of gene expression in treating osteosarcoma. miRNAs are small, non-coding RNA molecules that play critical roles in regulating gene expression by binding to the 3′ untranslated regions (UTRs) of target mRNAs, leading to their degradation or translational repression [212,213]. This regulatory function is particularly relevant in cancer, where the dysregulation of miRNAs can contribute to tumorigenesis, metastasis, and resistance to therapies [214,215].

In osteosarcoma, several studies have identified specific miRNAs that are either upregulated or downregulated, influencing tumor behavior and patient prognosis. For instance, miR-21 has been shown to promote cell invasion and migration by targeting the tumor suppressor gene RECK, thereby enhancing the metastatic potential of osteosarcoma cells [216,217]. Conversely, miR-140-5p has been identified as a tumor suppressor that inhibits cancer cell proliferation by downregulating GLUT-1, suggesting that restoring its expression could be beneficial in osteosarcoma treatment [218]. Additionally, the downregulation of miR-95-3p has been linked to suppressed cell growth and increased apoptosis in osteosarcoma cells, highlighting its potential as a therapeutic target [219].

Therapeutic delivery systems for miRNAs are crucial for their practical application in clinical settings. Nanoparticle-based delivery systems, such as dendritic polyglycerol nanopolyplexes, have been developed to enhance the stability and bioavailability of miRNAs in vivo [220]. These systems can facilitate the targeted delivery of miRNAs to tumor cells, thereby improving therapeutic outcomes. For example, the use of miR-200 family members has been explored for their ability to act as tumor suppressors by targeting genes involved in epithelial-to-mesenchymal transition (EMT), which is a critical process in cancer metastasis [220,221]. Moreover, the identification of circulating exosomal miRNAs in osteosarcoma has opened new avenues for non-invasive diagnostics and monitoring of treatment response [222]. The potential of miRNAs as biomarkers for osteosarcoma is underscored by studies showing that specific miRNAs correlate with disease progression and patient survival [223,224]. For instance, miR-26a downregulation has been associated with poor prognosis in osteosarcoma patients, indicating its role in tumor metastasis [223].

## 4. Immunotherapy in Osteosarcoma Treatment

### 4.1. The Tumor Immune Microenvironment (TIME)

Since the last decade, treatment modalities like chemotherapy and radiation used for cancer patients have moved towards antibody-based immunotherapies. Understanding TIME is essential for gauging the success of therapies, exploring the biomarkers, selecting the appropriate patient population, and finding new novel targets for therapies [225].

The tumor microenvironment is a composite of various cellular and extracellular matrices. It refers to the immune cells and their associated secretions within the tumor microenvironment, while microenvironment refers to the intercellular material and its humoral components [226]. The tumor-infiltrating immune cells (TIICs) are one of the critical constituents of tumor microenvironment [227]. Basic and clinical research on the tumor microenvironment, which consists of cancerous, stromal, and immune cells, demonstrates the critical role of antitumor immunity in cancer development and progression [228].

#### 4.1.1. Immune Cell Infiltration

Based on the immune cell infiltration, the TIME is generally described in two classes: infiltrated-excluded (I-E) and infiltrated-inflamed (I-I). In the former class, TIME is usually populated with immune cells. However, the tumor core could be relatively devoid of immune cells like cytotoxic lymphocytes (CTLs). This lack of CTL infiltration in some tumors is associated with the presence of tumor-associated macrophages (TAMs), which surround the tumor margins. The later infiltrated-inflamed (I-I) class has high infiltration of CTLs expressing PD-1 and leukocytes and tumor cells expressing the immune-dampening PD-1 ligand PD-L1 [225]. The knack of the organism to identify and eliminate abnormal cells, including cancerous cells, depends on its immunological system. Cancer cells have the immunogenicity that triggers an immune response. This characteristic of cancer cells is a key factor in the success of developing immunotherapeutic approaches [229]. The two most common immune cells infiltrating OS are macrophages and T lymphocytes [227].

#### 4.1.2. Prognostic Significance

I-E TIME is hypothesized as “cold” as these are poorly immunogenic. These cold tumors have CTLs with low expression of the activation markers GZMB (GRZB) and IFNG and poor infiltration of CTLs into the tumor core, making adaptive immunity less effective. Infiltrated-inflamed (I-I) TIMEs are considered to be immunologically ‘hot’ tumors. A subclass of I-I TIMEs called TLS-TIMEs, due to their cellular similarity with tertiary lymphoid structures (TLSs) like lymph nodes, having diverse lymphocytes, is created with a positive prognosis. The TIME evolves with tumor progression, altering immune cell composition and function, which impacts therapy effectiveness [225]. Poor CD8^+^ T cell infiltration is a negative prognostic marker associated with metastatic progression and worse outcomes [230].

### 4.2. Immune Evasion Mechanisms

A better understanding of immune evasion mechanisms, like avoiding recognition and killing by immune cells used by tumors, is crucial for combating cancers. Tumor-derived Extracellular Vesicles (EVs) are also implicated in the immune evasion mechanism [230]. A number of immune evasion strategies affect how precursor lesions progress into the invasive phase and aid in cancer’s progression from an early to a metastatic stage. By creating an immunosuppressive microenvironment and losing their immunogenicity or antigenicity, cancerous cells might evade the immune system’s destruction [229]. An essential stage in the development of cancer is immunoediting. There are three stages in this immunoediting process: “elimination”, “equilibrium”, and “escape”. White blood cells and tumor cells seem to have co-evolved. Cancer can occur under TCR-T cell therapy, possibly due to the loss of part of the HLA-A*02:01 allele in tumor cells after the loss of heterozygosity in tumor cells’ MHC. Furthermore, cells can reduce MHC-1 expression through autophagy involving NBR1. MHC-1 can be degraded selectively in the cell by lysosome. At this point, although MHC-1 expressions are downregulated, mutations are rarely detected at the gene level. Numerous studies have since demonstrated that most cancers that returned after CAR-T treatment either stopped expressing the CD19 gene or developed without it. Indoleamine 2,3-dioxygenase (IDO) has been steadily implicated in developing checkpoint inhibitor resistance in malignancies. T-cell failure due to overexpression of IDO provides an environment that is conducive to tumor cell growth and negates the effects of immune checkpoint inhibitors [226].

A mechanism called “cancer immunoediting” is one of the tumors’ immune evasion tactics. Antitumor immune responses triggered by antigen recognition in the tumor environment seem to result in cancer immunoediting. When the immune system interacts with cancer cells that have certain genetic alterations in the first place, the clones that have lost these mutations may proliferate selectively and biasedly, which could allow the tumor to evade the immune system.

It is possible that cancer cells do not evoke a strong exclusionary immune response for certain tumors because the immune system may not distinguish cancer cells that have lost particular antigens from healthy host cells. When costimulatory molecules are involved, the way mature APCs present antigens to T cells differs from how cancer cell antigens are presented to T cells. The simultaneous existence of a second signal from costimulatory molecules, like CD28, is necessary for the APCs to deliver antigens and trigger T-cell activation during antigen recognition. This second signal regulates the subsequent T-cell response. Antigen stimulation itself may be disregarded when the antigen is given to T cells without the second signal; this is known as unresponsive energy, and it causes the antigen-specific T cell responses to be lost. Even if T-cells are exposed to potent cancer-specific antigens produced from genetic alterations, cancer cells may not elicit T-cell responses because they lack crucial second signals [228].

### 4.3. Expression of Immune Checkpoint Molecules

Low expression of MHC-I and upregulation of immune checkpoints is another mechanism of immune evasion used by tumors. Impaired production of MHC class I/Human Leukocyte Antigen (HLA) class I antigens is one way cancers evade T cell-mediated immunosurveillance. For instance, in the EwS TME, HLA-G, and HLA-E, the non-classical MHC-I molecules implicated in the protective maternal–fetal barrier in the placenta are highly upregulated on tumor and myeloid cells [230]. Human autoimmune cells produce a class of receptor proteins called immune checkpoints, which can control the immune system’s activity level and help maintain immunological tolerance. Following immunoediting, tumors have also developed a mechanism that suppresses the function of immune cells by releasing signals through immunological checkpoints. ICI is responsible for preemptively inhibiting immunological checkpoints to enable immune cells to continue destroying the tumor. Currently, the most widely utilized inhibitors are PD-1, PD-L1, and CTLA-4 [226]. Immune checkpoint molecules such as programmed death 1 (PD-1) and cytotoxic T-lymphocyte-associated protein 4 (CTLA-4) were identified in 1990. To have anticancer effects, both compounds inhibit T cell activation. They serve as an organism’s defensive mechanism to stop excessive T cell activation and stop autoimmune reactions, and their expression rises in direct proportion to T cell activation. Ligands that attach PD-1 and CTLA-4 inhibit T-cell function. For instance, during antigen presentation, the costimulatory molecule CD80/CD86 produced on APCs binds to CD28 on T cells concurrently to increase T cell activation. On the other hand, active T cells produce CTLA-4, which competes with CD28 to block the costimulatory signal mediated by CD80/CD86 [228].

### 4.4. Immunotherapeutic Strategies

Understanding and modulating the TIME is essential for enhancing immunotherapy outcomes, such as immune checkpoint blockade (ICB) [225]. Upregulating MHC-I expression in pediatric sarcomas could be a viable tactic to trigger antitumor responses mediated by CD8^+^ T cells. This can be accomplished by activating proinflammatory pathways, such as type I IFNsIFNAR1/2-STAT1/2/3, type II IFN-IFNGR-STAT1, or TNF-TNF receptor-NF-κB. Targeting TAMs may be a promising therapeutic approach in several pre-clinical and clinical trials. These macrophages are tumorigenic as they help in angiogenesis, migration, extravasation, and chemotherapy resistance [230]. Augmentation of the body’s immune response is one of the promising strategies in cancer treatment. Modalities like cytokine therapy, adoptive T-cell transfer therapy, and antibodies that elicit both innate and adaptive immune responses are used in cancer immunotherapeutics [231]. Strategies to improve the effectiveness of immunotherapy will need to consider the immune escape mechanisms that cancer use. Chimeric antigen receptor (CAR) T-cell therapy, immune checkpoint inhibitors, and vaccinations are examples of immunotherapeutic approaches [229]. Alongside chemotherapy for treating osteosarcoma, immunotherapy has emerged as a promising avenue in which the body’s immune system recognizes and combats cancer cells. Recent immunotherapeutic strategies include immune checkpoint inhibitors, adoptive T cell therapy, and chimeric antigen receptor (CAR)-T cell therapy [232]. Cancer immunotherapies have been proposed as the fourth cancer treatment. In particular, the clinical application of immune checkpoint inhibitors, such as anti-CTLA-4 and anti-PD-1/PD-L1 antibodies, in various cancer types represents a breakthrough in cancer therapy [228]. The concept of immunotherapy has been acknowledged already for centuries. The development of innovative methods to use the immune system for cancer treatment was made possible by the quick rise in molecular biotechnology techniques. Due to their ability to produce long-lasting and successful therapeutic responses in cancer patients, a variety of strategies, such as adoptive cell treatments, monoclonal antibodies, checkpoint inhibitors, and oncolytic viruses, currently represent the most notable developments in cancer treatment [233].

CRISPR/Cas9 technology offers powerful tools for genetic engineering, targeting specific tumor characteristics in osteosarcoma. Its application in silencing genes such as CD44 has been demonstrated to reduce the metastatic potential of osteosarcoma cells, indicative of its potential to interfere with tumor progression [234]. CRISPR/Cas9 can induce precise genetic modifications that enable the development of more effective CAR T-cells by enhancing their specificity towards tumor cells while reducing off-target effects [235].

Moreover, CRISPR/Cas9 has been employed to identify synthetic lethal interactions in osteosarcoma, thus providing insights into vulnerabilities within this heterogeneous tumor type [236]. For example, studies suggest that targeting multiple pathways through gene editing might further synergize with CAR T-cell therapies, leading to a more effective treatment strategy [234]. The ability to modify the tumor microenvironment through specific gene knockouts or alterations can create a more conducive environment for CAR T-cell infiltration and activity [237].

The immunosuppressive TME in osteosarcoma is characterized by factors that inhibit T-cell function, including various immune cells, stromal components, and extracellular matrix proteins [238]. Several studies have shown that engineering CAR T-cells to express specific receptors or co-stimulatory molecules can enhance their ability to navigate such barriers. For example, employing a switchable CAR strategy could allow for temporal control over T-cell activity, thus enabling a more adaptable response to changing TME dynamics [239,240].

Additionally, the combination of CRISPR/Cas9 technology to modulate the expression of immunosuppressive factors within the TME presents a novel approach to facilitate CAR T-cell function. Targeting molecules that dampen T-cell responses, such as PD-1 ligands or other inhibitory receptors, may restore T-cell functionality and improve therapeutic outcomes [30,238].

### 4.5. Immune Checkpoint Inhibitors

Immune checkpoint blockers were the first antibody-based immunotherapy agents to be used. These act by blocking the receptor and ligand interaction of various molecules involved in cellular immunity [225]. Checkpoint blockade inhibitors, such as anti-programmed cell death protein 1 and anti-cytotoxic T-lymphocyte-associated antigen-4, chimeric antigen receptor T-cell therapy, and monoclonal antibodies, are effective strategies in treating some types of cancers [231,241,242]. These drugs stimulate the immune system to fight cancer cells and have revolutionized cancer treatment by increasing survival in a range of cancer types. Since 2011, many ICIs have been available in the market for treating cancers. T-cells of the adaptive immune system are the focus of immune checkpoint therapy because of their capacity to identify particular antigens [243]. Immune checkpoint inhibitors demonstrate that these are not always effective but are instead only effective in limited cancer populations [228].

#### 4.5.1. Immune Checkpoint Inhibitors and Osteosarcoma

The landscape of immunotherapy for osteosarcoma is evolving, with multiple strategies being explored to enhance the effectiveness of immune-based treatments. Several studies highlight a multifaceted immunotherapy approach for osteosarcoma, emphasizing the potential of checkpoint inhibition, targeted CAR-T modifications, and combinational strategies to improve patient outcomes.

#### 4.5.2. CAR T-Cell Therapy Targeting Osteosarcoma Antigens

Inducing CXCL10 expression in the EwS TME in order to enhance T cell infiltration may, thus, be a promising therapeutic strategy [230]. Chimeric antigen receptor (CAR)-T cell therapy is a novel form of immunotherapy derived from adoptive T cell transfer therapy. The potential targets for CAR-T cell therapy in osteosarcoma are tumor-associated antigens. These are expressed in both normal and tumor tissues but are more highly expressed in tumor tissues. Many potential targets for CAR-T cell therapy in osteosarcoma include receptor tyrosine kinases (HER2, IGF1R, ROR1, and EphA2), cell surface glycoproteins (CSPG4, FRα, FRβ, and EC17), B7-H3 (CD276), disialoganglioside (GD2), natural killer group 2D (NKG2D), activated leukocyte cell adhesion molecule (ALCAM/CD166), interleukin-11 receptor alpha (IL-11Rα), and fibroblast activation protein (FAP) [30]. In therapy with TCR/CAR-T cells, enough high-quality antigen-responsive T cells extracted from tumor tissues in a given patient may determine the availability and efficacy of TIL treatment. Peripheral blood mononuclear cell (PBMC)-derived lymphocytes, which synthetically express a desired TCR or chimeric antigen receptor (CAR), have been developed and used in clinical settings as a new T cell treatment to overcome these restrictions. T cells that have been transduced with antigen-specific TCRs are used in TCR-T therapy. The process of delivering T cells with a *CAR* gene, which comprises gene fragments from intracellular TCR domains, a fragment from a cancer antigen-recognizing antibody gene, and other T cell costimulatory molecules, is known as CAR-T therapy [228].

One promising approach involves a clinical trial using multi-antigen stimulated cell therapy (MASCT-I), which was evaluated in combination with camrelizumab (a PD-1 inhibitor) and apatinib (a VEGFR2-targeting TKI) in patients with advanced sarcomas. This pilot study demonstrated that the combination therapy was safe and showed promising efficacy, particularly in osteosarcoma patients, where the objective response rate (ORR) reached 33.3% and the median progression-free survival (PFS) was 5.7 months [244].

Meanwhile, CAR-T cell therapy targeting GD2, a surface antigen expressed in osteosarcoma, was investigated in a phase I trial, which revealed that while GD2 CAR-T cells were safe and feasible, their clinical efficacy was limited, with most patients experiencing only stable disease before eventual progression. The study identified key immune determinants influencing CAR-T expansion, including the presence of naïve and memory T cells in pre-treatment samples and a correlation between CXCR3^+^ monocytes and enhanced CAR-T expansion [245].

A more advanced switchable CAR-T strategy was also explored to improve CAR-T effectiveness against osteosarcoma. This approach utilized anti-FITC CAR-T cells activated by an anti-B7-H3-FITC monoclonal antibody, enabling tumor-specific targeting and greater control over CAR-T activation. In preclinical models, this system effectively redirected CAR-T cytotoxicity against osteosarcoma tumors, showing enhanced infiltration and antitumor activity in the presence of the switch molecule [239]. It is important to remember that only a small percentage of individuals receiving immunotherapy have desirable therapeutic outcomes. Specifically, solid tumors typically have a tumor microenvironment that suppresses T-cell activation and promotes tumor growth. Cytokine storm and autoimmune events are noteworthy adverse effects of such treatment [233]. Therapeutic targeting of PD-L1 and PD-1 immune checkpoints, which are expressed in ~20% of pediatric sarcoma patients in EwS and OS, has not shown clinical efficacy. However, OS tumors that score in the top quartile of immune infiltration may benefit from ICB. The histological classification of sarcomas affects the clinical response to ICB due to dynamic and variable expression of immune checkpoints [230].

One of the most commonly reported immune-related toxicities following CAR T-cell therapy is cytokine release syndrome (CRS). This condition is characterized by the rapid release of cytokines into the bloodstream following T-cell activation, which can lead to symptoms ranging from mild flu-like manifestations to severe, life-threatening complications such as hypotension and acute respiratory distress syndrome [246,247]. CRS is particularly prominent in therapies targeting high antigen loads, which can elicit a robust T-cell response, thereby amplifying cytokine secretion [30,248]. Additionally, immune effector cell-associated neurotoxicity syndrome (ICANS) is another significant complication, manifesting as neurological symptoms ranging from confusion to seizures and, in severe cases, cerebral edema [249,250].

Beyond CRS and ICANS, CAR T-cell therapy can induce “on-target, off-tumor” toxicity. This occurs when CAR T cells, directed against specific antigens, also inadvertently affect normal tissues expressing the same antigens, leading to adverse effects on organs where the targeted antigen is normally present, which can result in additional complications such as organ dysfunction or inflammatory responses in healthy tissues [251,252]. For instance, targeting the CD19 antigen has been linked to damage to normal B cells and subsequent hypogammaglobulinemia, predisposing patients to infections [253]. Specific concerns in osteosarcoma therapy include the risk of damage to surrounding healthy tissues due to the high expression of similar antigens in those tissues. Research suggests that careful selection of target antigens is vital, given the limited tumor-specific targets available for osteosarcoma [254].

Strategies are being explored to mitigate these toxicities, exemplified by the development of safety switches and suicide genes in CAR constructs to enhance the safety profiles of these therapies [248,254]. By addressing these immune-related toxicities, researchers aim to expand the therapeutic window of CAR T-cell therapy for osteosarcoma and other solid tumors, ultimately improving patient outcomes and reducing adverse effects.

Despite being very promising, the low response rates of checkpoint inhibitors in osteosarcoma can be attributed to several interrelated factors, including the tumor microenvironment, immune evasion mechanisms, and the intrinsic properties of osteosarcoma itself. Firstly, osteosarcoma exhibits a relatively low immunogenicity compared to other malignancies, which contributes to its diminished response to immune checkpoint inhibitors (ICIs). Specifically, the tumor microenvironment in osteosarcoma is characterized by a deficiency in immune cell infiltration, particularly tumor-infiltrating lymphocytes (TILs), and the presence of immunosuppressive cells such as myeloid-derived suppressor cells (MDSCs) and regulatory T cells (Tregs) [255,256,257]. This immunosuppressive environment limits the activation and efficacy of T cells, which are necessary for ICI function, as TILs often exhibit high expression of immune checkpoint molecules like PD-1, further impairing their antitumor activity [258,259]. Research has shown that the expression levels of various immune checkpoints in osteosarcoma, including PD-L1, are crucial in determining the potential effectiveness of checkpoint blockade therapies [260].

Moreover, the heterogeneity of osteosarcoma and its complex biology complicate therapeutic responses. Although there is evidence of cytotoxic T-cell infiltration and promising immune responses in some cases, the overall response rates remain disappointingly low, often falling below 10% [261,262]. The existence of “cold” tumors, which lack sufficient immune cell presence, adversely affects the likelihood of achieving effective antitumor responses with single-agent therapies [263]. In fact, studies indicate that the optimization of the immune landscape, possibly through combination therapies or alternative immunotherapeutic strategies, is necessary to improve the clinical efficacy of ICIs in this cancer type [264,265,266]. A critical factor contributing to the unresponsiveness of osteosarcoma to ICIs is the persistence of signaling pathways that facilitate immune evasion. Osteosarcoma cells can exploit various mechanisms to dampen immune responses, including the overexpression of checkpoint molecules and secretion of immunosuppressive cytokines [267,268].

### 4.6. Adoptive Cell Therapy

Immune checkpoint therapy targets T-cells of the adaptive immune system due to their ability to recognize specific antigens [243]. To improve the targeted lethality, adoptive cell therapy involves obtaining the patient’s immune cells, growing and amplifying them in vitro, and reintroducing them into the patient. The benefit is that the patient can prevent immunological rejection of foreign cells or antibodies, and the target cells can be eliminated as precisely as with prior immunotherapy. Depending on the cells collected and cultivated, therapies can also be categorized as TCR-T cell treatment, CAR-T cell therapy, lymphokine-activated killer cell therapy, natural killer (NK) cell therapy, etc. The T cells isolated from the patient’s body undergo gene editing for the expression of the new generation of T cells to detect the TCR of cancer cells and more precisely and effectively identify the tumor. T cells are then cultivated and given back to the patient [226].

#### Natural Killer (NK) Cell Therapy

Natural killer (NK) cells are lymphocytes that may identify cancerous cells by balancing the detection of cell-surface stress and danger markers. NK cells release cytokines and use a variety of methods to cause target cell lysis after being triggered by such recognition. As key mediators of immunotherapeutic modalities such cytokines, antibodies, immunomodulating medications, and stem cell transplantation, NK cells are becoming more widely acknowledged for their function in regulating tumor growth and metastasis [269]. In the absence of major histocompatibility complex (MHC)-restricted receptor–ligand interactions, NK cells can identify a range of stressed cells, including cancer cells. NK cells are crucial mediators of various therapeutic interventions and play a part in regulating the growth and spread of tumors. The fundamental idea behind adoptive natural killer (NK) cell therapy is that adoptive transfer of NK cells can restore a natural deficit in innate immunity, which is caused by a combination of immunosuppressive mechanisms that result in reduced function and cancer-induced decreases in NK cell numbers. The fundamental idea behind adoptive natural killer (NK) cell immunotherapy is that adoptive transfer of NK cells can restore a natural defect in innate immunity, which is caused by a combination of immunosuppressive mechanisms that result in suppressed function and cancer-induced decreases in NK cell numbers [270].

### 4.7. Cancer Vaccines

The creation of the HPV vaccination has opened the door for the creation of additional cancer vaccines [231]. The goal of therapeutic cancer vaccines is to induce tumor regression, eradicate minimal residual disease, establish lasting antitumor memory, and avoid non-specific or adverse reactions [271]. The clinical research indicated that patients with particular infectious diseases had a low cancer incidence. This served as the foundation for developing this cancer vaccine. This might be explained by how inflammation and infection expose antigens that cancer cells aberrantly produce. It could also be a secondary effect, in which the cancer cells are impacted by immunological memory accumulated from previous inflammation or infection. Similarly, the incidence of ovarian cancer is reduced by antibodies against aberrant cell surface-associated mucin (MUC1) generated after a mump infection. Additionally, Bacillus Calmette–Guerin has long been utilized as a vaccine against tuberculosis and is currently being used extensively as a therapeutic vaccination against bladder cancer [228].

#### Development of Peptide-Based and Dendritic Cell Vaccines

The ability of dendritic cells to initiate immunity and manage and modulate the sort of immune response has made them particularly appealing as vehicles for mRNA delivery in immune treatment techniques. Research has focused on creating an ex vivo population of antigen-loaded dendritic cells capable of inducing strong and enduring CD8^+^ and CD4^+^ T-cell responses in cancer patients. Nevertheless, the generation of immunopotent dendritic cells ex vivo for efficient antitumor immune responses cannot be completely replicated [272]. There are various issues related to the development of the Cancer Vaccines. Test designs may have been defective when several traditional cancer vaccines were being developed for clinical use. For instance, patients with a terminal diagnosis whose immune systems were already significantly weakened by exposure to multiple medical therapies, including surgery, chemotherapy, and radiation therapy, or by the advancement of the illness, were frequently used to assess the clinical effects of cancer vaccines. To use vaccines and achieve the desired outcomes in clinical settings, it is also crucial to create auxiliary technologies such as adjuvants, production processes, and delivery systems. Current medication laws may make it more difficult to introduce such cutting-edge technology [228].

### 4.8. Oncolytic Viruses

Oncolytic viruses serve as a model to demonstrate their versatile nature and show how they can complement other cancer therapies to gain optimal patient benefits. The use of oncolytic viruses in treating cancer can bring cancer immunotherapy to the next higher level [233]. Viruses due to their selective replication and induction of cytopathic effects are considered suitable for cancer immunotherapy [273].

#### Mechanism and Therapeutic Potential

Oncolytic viruses can make tumors immunogenic that the immune system does not appear to recognize. The use of oncolytic viruses in altering the tumor microenvironment and enabling T-cell treatments to function in solid tumors may be an additional strategy worth investigating. The cancer cells are infected and lysed by an oncolytic virus. Oncolytic viruses are either naturally occurring or can be created in a lab by altering naturally occurring viruses. These viral changes ushered in a new age of less harmful virus-based treatments for cancer. The virus fiber knob attaches itself to receptors on the surface of tumor cells during the early stages of viral infection. Depending on the virus’s serotype, distinct receptors mediate this interaction. Serotype 3 adenoviruses bind desmoglein-2, CD46, or CD80/86, but serotype 5 adenoviruses primarily bind to the coxsackie and adenovirus receptor (CXADR). While some receptors, like CXADR, are regrettably downregulated in many advanced cancers, others are commonly present in cancer cells. Later, the virus internalizes due to contact between its penton proteins and the integrins of tumor cells. Ultimately, the virus DNA enters the nucleus by a series of steps, where transcription of the early viral proteins (E1–E4) begins. Thousands of new viral progeny arise following late protein expression, break the cell membrane a few days later, and infect other cells until the immune system finally halts the process. Multiple tumor-associated antigens are revealed by immunogenic cell death and presented to the immune system by activated mature dendritic cells [233]. These viruses stimulate T-cell responses, leading to antitumor immunity. Hence, these are considered immunogenic in nature and can be used to start an antitumor immune response. Tumor cells are relatively more susceptible to viral infections than normal cells. Therefore, the viruses target and destroy tumors. Oncolytic viruses stimulate the effector cells by infecting tumor cells, as well as changing the TME from cold to hot. Direct tumor lysis occurs due to infection, which leads to the release of DAMPS and PAMPS. These are recognized by immune subsets like DCs, NK cells, and so on due to their PRR expression. The inflammatory cytokines are released, which attract other immune cells. The viral replication in cancer cells leads to the expression of TAA. The DC captures TAA and presents these to T cells, which help recruit T cells in tumors causing their ICD. Positive therapeutic results are expected for patients in any CAR-T therapy, checkpoint inhibitors, or cancer vaccines combined with oncolytic viruses. This is due to their role in the production of TAA and TIL [273].

## 5. Novel Therapeutic Approaches and Clinical Trials

Osteosarcoma (OS) treatment has evolved significantly, with a focus on innovative therapeutic strategies that aim to improve clinical outcomes and address the challenges of chemotherapy resistance [15]. These strategies include targeting molecular mechanisms of treatment efficacy and resistance [14], exploring epigenetic therapies, and utilizing gene editing technologies [274,275]. Personalized medicine approaches, such as genomic profiling, are being developed to tailor treatments based on individual patient characteristics [276]. These innovative strategies aim to transform OS treatment and improve patient survival rates.

### 5.1. Epigenetic Therapy

Epigenetic therapies, including DNA methyltransferase inhibitors (DNMTi) and histone deacetylase (HDAC) inhibitors, have shown promise in treating osteosarcoma (OS) by modifying gene expression without altering DNA sequences [277]. DNMTi can restore the expression of tumor suppressor genes (TSG) silenced by hypermethylation, while HDACi enhance gene expression through histone modification, potentially improving sensitivity to conventional chemotherapeutics [278]. For instance, DNMTi like MC3343 have shown promising findings in OS treatment. MC3343 blocks tumor proliferation and induces osteoblastic differentiation, differing from the conventional FDA-approved nucleoside inhibitor 5-azacytidine (5azadC). Furthermore, MC3343 exhibits synergistic effects when combined with doxorubicin and cisplatin [279].

Studies have demonstrated that HDACi, such as AR-42 and panobinostat, are potent inhibitors of OS cell viability and can synergize with standard chemotherapeutic agents like doxorubicin [280]. AR-42 exhibited greater potency than SAHA in inducing apoptosis in osteosarcoma cells. Furthermore, the combination of AR-42 and doxorubicin resulted in a synergistic effect on inhibiting cell viability. Both AR-42 and SAHA triggered apoptosis via the mitochondrial pathway, but AR-42 was more effective at inhibiting pro-survival Akt signaling [281]. While HDACi monotherapy has shown limited success in clinical trials, combination regimens appear more promising [282,283]. Table 1 summarizes DNMTi and HDACi along with their roles in OS treatment.

### 5.2. Gene Therapy

Gene therapy approaches, particularly CRISPR/Cas9 and Antisense Oligonucleotides (ASOs), offer exciting new pathways for targeting oncogenes, overcoming chemoresistance, and improving patient outcomes. CRISPR/Cas9 technology has emerged as a potential strategy for correcting osteosarcoma (OS) mutations. Research indicates that CRISPR/Cas9 can effectively silence oncogenes, such as CD44, which is implicated in the metastatic behavior of osteosarcoma cells. In vitro studies have demonstrated that targeting CD44 with CRISPR/Cas9 significantly reduces its expression, leading to decreased cell proliferation and migration in osteosarcoma cell lines [290]. Furthermore, a CRISPR-Cas9-mediated CDK11 knockout has demonstrated reduced cell proliferation, viability, migration, and invasion in OS cell lines [291]. Moreover, knocking out the ABCB1 gene using CRISPR-Cas9 technology restored sensitivity to doxorubicin in the treated OS cells, highlighting the potential of CRISPR-Cas9 as a therapeutic strategy to overcome drug resistance [292]. Recent advances in CRISPR/Cas9 technology have shown promise for correcting mutations in cancer-related genes like TP53 and RB1, which are frequently altered in osteosarcoma [293].

Antisense oligonucleotides (ASOs) represent another innovative strategy for targeting oncogenes and resistance genes in osteosarcoma. ASOs are short synthetic RNA or DNA molecules that bind to complementary nucleic acid sequences, modulating protein expression through various mechanisms [294,295]. In osteosarcoma, ASOs have shown potential in targeting drug resistance-related genes, such as FOXC2-AS1, which promotes doxorubicin resistance [296]. Furthermore, ASO targeting the insulin-like growth factor (IGF) signaling pathway has shown potential in preclinical models, suggesting that ASOs could enhance the efficacy of existing chemotherapeutic agents [297]. However, challenges remain in ASO delivery, stability, and cellular uptake [298].

Despite these advancements, several challenges remain in implementing gene therapy and novel agents in clinical practice. The heterogeneity of osteosarcoma tumors, coupled with the complexity of their genetic profiles, complicates the identification of suitable targets for therapy [299]. Moreover, the delivery mechanisms for gene therapies require optimization to ensure effective uptake by tumor cells while minimizing off-target effects [300]. To address these challenges, researchers have developed tumor-targeted delivery systems [236] and synthetic switches to self-regulate Cas9 expression [301]. Additionally, DNA nanotechnology-based approaches leveraging collaborative effects show the potential to reduce hybridization-dependent off-target effects [302].

### 5.3. Personalized Medicine Approaches

Personalized medicine approaches are emerging as promising strategies for treating osteosarcoma (OS) and overcoming chemoresistance. These approaches leverage genomic profiling, patient-specific targeting, and pharmacogenomics to develop customized therapeutic regimens. Genomic profiling of OS has revealed significant heterogeneity across patients, highlighting the need for personalized treatment strategies. Whole-genome sequencing (WGS) of patient tumors can identify somatic copy number alterations (SCNAs) and structural rearrangements that may drive tumor growth. These findings can be used to identify patient-specific therapeutic targets [303]. This personalized approach enhances therapeutic success and minimizes unnecessary exposure to ineffective treatments. For instance, the identification of specific biomarkers can guide the use of novel agents, such as immune checkpoint inhibitors, by targeting proteins like PD-1, PD-L1, and CTLA-4, which have shown promise in preclinical models of osteosarcoma [304,305,306]. Drug resistance remains a significant challenge, necessitating the identification of biomarkers for patient stratification and targeted treatments [307].

Pharmacogenomics is crucial in understanding how genetic variations affect drug metabolism and response in osteosarcoma (OS) patients. Studies have identified several genetic variants associated with treatment efficacy and toxicity of chemotherapeutic agents like doxorubicin and cisplatin [308,309]. For instance, polymorphisms in DNA repair and drug metabolism genes have been associated with treatment response and toxicity in osteosarcoma patients receiving MAP (methotrexate, doxorubicin, cisplatin) chemotherapy. Studies have linked ERCC2/XPD rs1799793 and ABCC2 rs2273697 variants to poor pathological response [310], while the ERCC1 rs11615 CC genotype was associated with better chemotherapy response and improved overall survival [93,311]. Furthermore, ABCC2 rs3740066 and ABCB1 rs1128503 variants were linked to reduced myelotoxicity and increased methotrexate levels, respectively [312]. Variants in ABCC1, ABCC3, and SLC22A1 genes have been associated with drug resistance and metastasis [313]. Identifying such pharmacogenetic markers could help tailor treatment to improve efficacy and reduce toxicity in osteosarcoma patients [314]. Personalized dosing strategies considering these genetic factors can optimize therapeutic outcomes and reduce adverse effects. Research shows that adjusting chemotherapy dosages based on pharmacogenomic data enhances treatment efficacy while minimizing toxicity [13]. A summary of the clinical trials is presented in Table 2.

### 5.4. New Therapies, Challenges, and Limitations

Bringing new therapies, such as immunotherapy and other innovative treatments, to osteosarcoma patients involves a myriad of real-world challenges, including high costs, regulatory approvals, and accessibility issues. Osteosarcoma, a highly aggressive malignancy that predominantly affects young individuals, currently presents significant therapeutic challenges, necessitating the exploration of novel biological agents and therapeutic strategies [33,322,323]. Immunotherapy, while promising, is often associated with high treatment costs, which can constitute a substantial barrier for patients, families, and healthcare systems.

The financial burden of advanced treatment protocols, such as immune checkpoint inhibitors and monoclonal antibodies, can lead to significant disparities in access, especially in pediatric populations, where the financial implications may impact family stability [324]. Furthermore, high-cost therapies may not be adequately covered by insurance, significantly limiting patient options [325]. The development and implementation of novel treatments also necessitate extensive research funding, further driving up costs prior to market entry [183]. The pathway to regulatory approval for new cancer therapies is intricate and often lengthy. Novel treatments, particularly in the context of immunotherapy, require rigorous preclinical and clinical testing to demonstrate safety and efficacy. Regulatory bodies, such as the FDA and EMA, necessitate comprehensive data sets, which can prolong the timeline for patient access [326,327]. For instance, mifamurtide is an approved therapy in Europe for osteosarcoma, based on a study showing improved overall survival when used with conventional chemotherapy [323]. This intensive regulatory scrutiny can delay the introduction of potentially life-saving therapies, leaving many patients without viable treatment options during crucial phases of their care [328]. Even when new therapies receive regulatory approval, accessibility remains a pressing concern. Availability may be geographically limited, particularly for patients in rural or underserved areas, further exacerbating health disparities [16]. The integration of novel immunotherapies often requires specialized facilities equipped with the necessary expertise and resources, which may not be universally available [329]. For patients with metastatic osteosarcoma, whose prognosis can change rapidly, delays in accessing cutting-edge treatments can significantly affect their survival outcomes. The transition from research to clinical application of new therapies for osteosarcoma is fraught with significant barriers. The challenges of high costs, rigorous regulatory pathways, and accessibility issues must be navigated to realize the benefits of these therapies for patients in need. Addressing these barriers requires a collaborative effort among researchers, healthcare providers, policymakers, and pharmaceutical companies to promote equitable access to innovative treatments in osteosarcoma therapy.

## 6. Summary and Future Directions

### 6.1. Summary of Therapeutic Advancements

Osteosarcoma remains a formidable challenge, particularly for children and adolescents who often endure physically and emotionally taxing treatments. Over the years, researchers and clinicians have worked tirelessly to break through the barriers posed by chemotherapy resistance. Their efforts have led to promising strategies that target critical resistance mechanisms such as drug efflux transporters, altered drug metabolism, and boosted DNA repair pathways to restore tumor sensitivity and improve patient outcomes. Alongside refinements in conventional chemotherapy, the field has made great strides in exploring targeted therapies that directly focus on key molecular alterations, like aberrant receptor tyrosine kinases and dysregulated PI3K/Akt/mTOR signaling. At the same time, immunotherapy has brought hope by harnessing our innate defenses, exemplified by checkpoint inhibitors, CAR T-cell innovations, and oncolytic viruses. These advancements offer patients and families newfound optimism for more effective, less toxic treatments.

The integration of targeted therapy, immunotherapy, and nanotherapy presents a potential paradigm shift in the treatment of osteosarcoma, particularly in addressing the challenges posed by tumor heterogeneity and metastatic disease. Osteosarcoma, a highly aggressive bone tumor, has a well-documented resistance to conventional chemotherapy, necessitating innovative approaches to enhance treatment efficacy and patient outcomes [330]. Nanotechnology presents a novel approach for enhancing drug delivery and overcoming the challenges associated with systemic treatments. Nanoparticles can be engineered for targeted delivery, thus enhancing the concentration of therapeutic agents directly at tumor sites while minimizing systemic toxicity. Recent studies have proposed the use of hyaluronic acid-based nanoparticles to deliver chemotherapeutics alongside immunomodulators, leveraging their ability to modify the immunosuppressive tumor milieu [331]. This synergistic approach is critical in enhancing the penetration of chemotherapeutic agents, ensuring effective tumor cell destruction while fostering an environment favorable for immune system activation [331].

Furthermore, the incorporation of targeted nanotherapy could be particularly beneficial when combined with genetic profiling, allowing for personalized treatment regimens based on individual tumor characteristics [332]. This direction aligns with ongoing advances in genome-informed therapies, which aim to tailor therapeutic interventions to the unique molecular landscape of each patient’s tumor, potentially leading to improved response rates and survival outcomes [254].

### 6.2. Future Perspectives

Advancing osteosarcoma therapy requires sustained research efforts and robust clinical trials to refine novel agents and integrate them effectively with existing treatments. Ongoing work to identify reliable biomarkers will help match patients with personalized interventions, such as specific molecular inhibitors or immunotherapeutic regimens tailored to tumor genetics. Just as essential is a multidisciplinary collaboration among researchers, clinicians, and the pharmaceutical industry, ensuring a constant exchange of scientific insights and resources necessary to translate preclinical discoveries into clinically actionable therapies. This synergy will be vital for validating combination approaches such as targeted drugs plus immunotherapy and fine-tuning drug delivery systems like nanoparticles to optimize tumor-specific drug accumulation.

### 6.3. Call to Action

Despite progress, osteosarcoma therapy still faces persistent challenges, underscoring the urgency for innovative strategies. Researchers must continue exploring underappreciated resistance mechanisms, including epigenetic alterations and cancer stem cell populations, to open new therapeutic windows. Clinicians, meanwhile, should adopt a multidisciplinary mindset integrating surgical, pharmacological, and immunological expertise to offer patients comprehensive, individualized treatment plans. Finally, partnerships with industry are essential for expediting drug development and ensuring that cutting-edge therapies reach patients safely and efficiently. We can only continue to improve survival outcomes and quality of life for individuals battling osteosarcoma through collective, concerted efforts.

## Figures and Tables

**Table 1:** Overview of DNMT inhibitors and HDAC inhibitors and their roles in osteosarcoma treatment.

Drug Type	Epigenetic Approach	Study Type	Treatment Protocol	Treatment Response	Synergistic Effects	Ref.
Decitabine (5-aza-2′-deoxycytidine)	DNA Methyltransferase (DNMT) inhibition	In vitro cell line study	Decitabine alone or in combination with chemotherapy (doxorubicin or cisplatin)	Low potency as a single agent	Synergistic with cisplatin in high-DNMT1-expressing cells, while doxorubicin showed consistent effects across all cell lines	[284]
MC3343	DNA Methyltransferase (DNMT) inhibition	In vitro (cell lines) and in vivo (patient-derived xenograft model)	MC3343 alone or in combination with chemotherapy (doxorubicin or cisplatin)	Inhibits cell proliferation, induces osteoblastic differentiation, cytostatic in vivo	Synergistic effects with doxorubicin and cisplatin	[279]
5-Azacytidine	DNA Methyltransferase (DNMT) inhibition	In vitro cell line study	5-Aza-dC (2.5 µM) alone or combined with TSA (300 nM)	Induced growth arrest and reprogramming of MDR OS cells	Synergistic effects with TSA (HDAC inhibitor)	[285]
Zebularine (Zeb)	DNA Methyltransferase (DNMT) inhibition	In vitro cell line study	Zebularine (1, 10, or 100 μM) for 48 h.	Inhibits cell growth, cytotoxic effects	Synergistic effects with SAHA (HDAC inhibitor)	[286]
LBH589 (Panobinostat)	Histone Deacetylase (HDAC) inhibition	In vitro (cell lines) and in vivo (mouse xenograft model)	Continuous exposure to LBH589 (0.5–500 nM in vitro, 2–10 mg/kg in vivo)	Induced differentiation and senescence; sustained cytostatic response in vivo	N/A	[287]
Trichostatin A (TSA)	Histone Deacetylase (HDAC) inhibition	In vitro cell line study	TSA treatment with varying concentrations (0–200 nM)	Inhibition of cell growth Induced apoptosis, increased p53 acetylation.	N/A	[288]
Romidepsin	Histone Deacetylase (HDAC) inhibition	In vitro and in vivo preclinical study	In vitro: Dose range of 5.9–150 nM in cell lines. In vivo: Administered subcutaneously (2.4 mg/kg) twice weekly	Reduced lung metastatic growth and improved survival	N/A	[289]
SAHA (Vorinostat)	Histone Deacetylase (HDAC) inhibition	In vitro cell line study	SAHA (1, 5, or 10 μM) for 48 h.	Inhibits cell growth, cytotoxic effects	Synergistic with Zebularine (DNMT inhibitor)	[286]
AR-42	Histone Deacetylase (HDAC) inhibition	In vitro cell line study	AR-42 (0.1–10 μM) for 24–72 h	Induced apoptosis, reduced cell viability	Synergistic with doxorubicin	[281]

N/A: Not Applicable (no synergistic effects reported).

**Table 2:** Clinical Trials for Osteosarcoma: Novel Treatment Strategies and Outcomes.

Clinical Trial Identification	Study Phase	Population	Treatment Protocol	Primary Outcomes	Novel Strategy	Type of Targeted Therapy	Ref.
NCT04661852	Phase I study	Relapsed osteosarcoma and Ewing sarcoma patients (n = 12)	Cabozantinib + topotecan + cyclophosphamide	Safety and toxicity	Multi-agent targeted therapy	Receptor tyrosine kinases (RTKs) inhibitor	[315]
NCT02406781	Phase II study	Patients with advanced osteosarcomas (n = 17)	Pembrolizumab + metronomic cyclophosphamide	Objective response rate and 6-month non-progression rate	Immunotherapy + chemotherapy	PD-1 inhibitor (Pembrolizumab)	[316]
NCT00631631	Phase III trial	Patients < 30 years with metastatic osteosarcoma (n = 91)	L-MTP-PE + standard chemotherapy vs. chemotherapy alone	Event-Free Survival (EFS) and Overall Survival (OS)	Immunotherapy combined with standard chemotherapy	N/A	[317]
NCT01650090	Phase Ib/IIa study	Recurrent osteosarcoma patients with pulmonary metastases (n = 19)	Inhaled lipid cisplatin (ILC)	Safety and efficacy	Novel delivery of Cisplatin (Inhaled Lipid)	N/A	[318]
NCT04417062	Phase II trial	Patients aged 12–40 with recurrent osteosarcoma	Olaparib + ceralasertib	4-month event-free rate (Cohort 1), tumor sample submission (Cohort 2)	Dual inhibition of DNA repair pathways	PARP inhibitor (Olaparib), ATR inhibitor (Ceralasertib)ibitors	[319]
NCT04690231	Phase II study	Patients aged 2–25 years with refractory or relapsed osteosarcoma (n = 33)	Apatinib (500 mg/day) + Ifosfamide and Etoposide (IE)	Objective responses, event-free survival, overall survival	Combination of TKI with chemotherapy	Multi-kinase inhibitor (Apatinib)	[320]
NCT04294511	Phase II trial	Resectable osteosarcoma patients (n = 75)	Camrelizumab + doxorubicin/liposomal doxorubicin + cisplatin + methotrexate + ifosfamide	Rate of good tumor necrosis (TNR ≥ 90%)	Combination of immunotherapy and multiple chemotherapeutics	PD-1 inhibitor (Camrelizumab)r	[321]

N/A: Not Applicable.

## Data Availability

Not applicable.
